# Supplementary figures and images for: Multi-step pathway engineering in probiotic Saccharomyces boulardii for abscisic acid production in the gut
Source: Metab Eng Commun. 2025 May 30;20:e00263. doi: 10.1016/j.mec.2025.e00263 (PMC12173631; doi:10.1016/j.mec.2025.e00263)

A

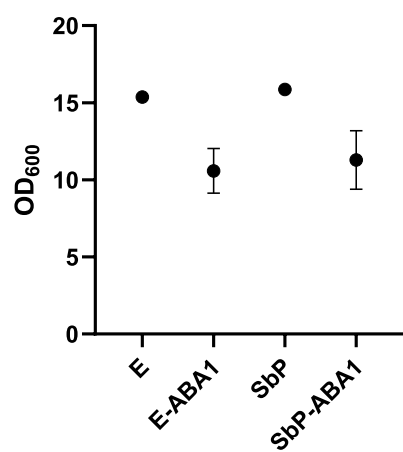

B

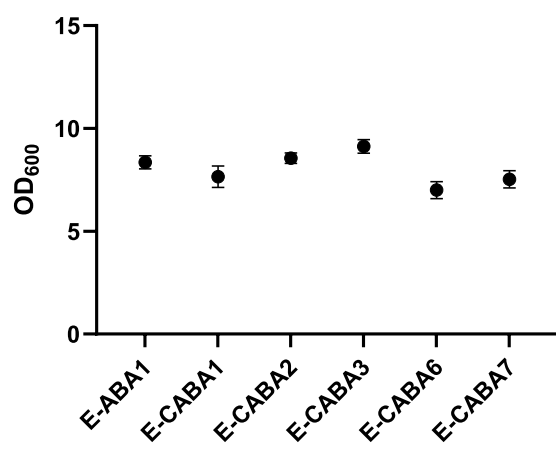

C

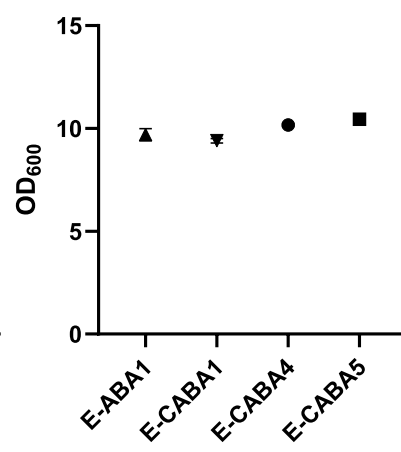

Supplement: Multimedia component 4 [file mmc4.pdf]

**A**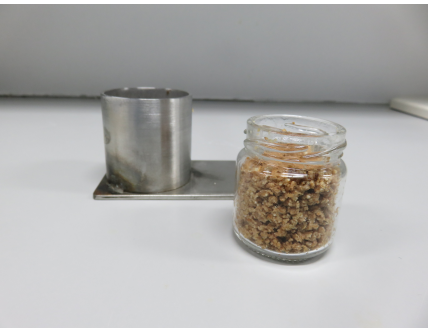**B**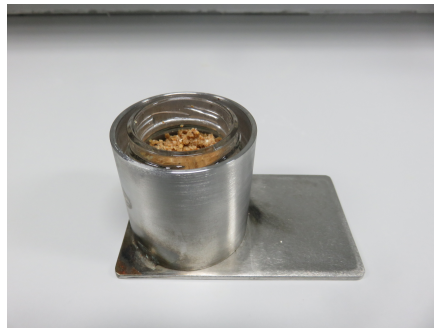**C**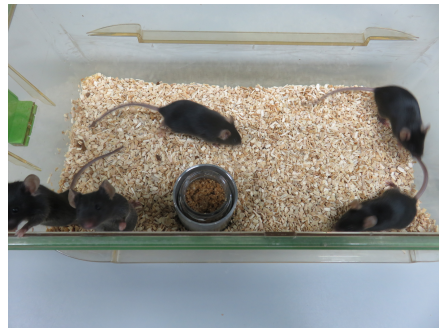

Supplement: Multimedia component 5 [file mmc5.pdf]

A

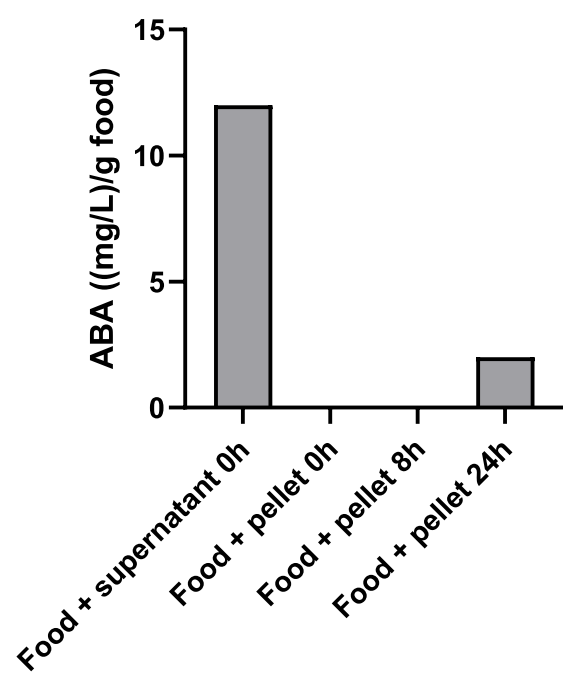

B

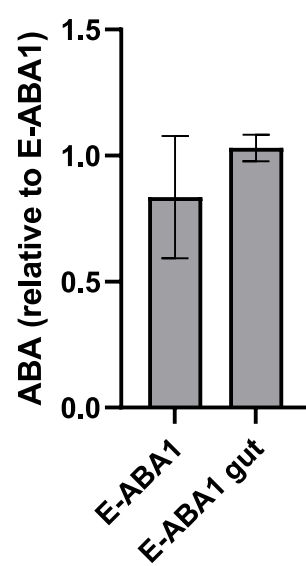

Supplement: Multimedia component 6 [file mmc6.pdf]

A

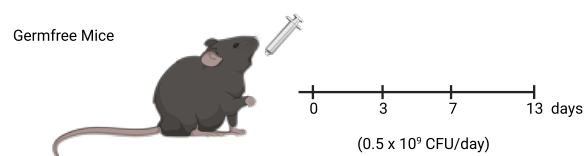

B

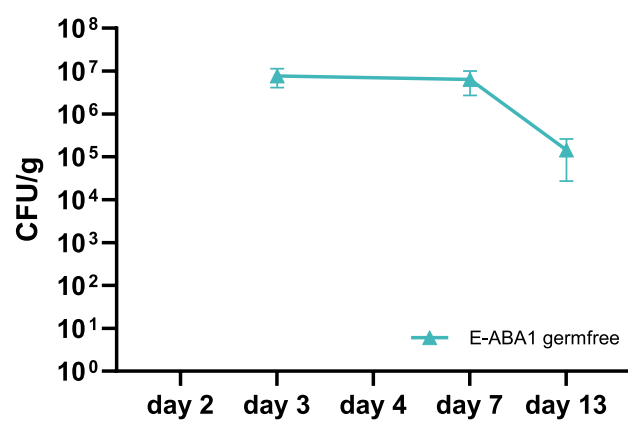

Supplement: Multimedia component 7 [file mmc7.pdf]

A

TABA3

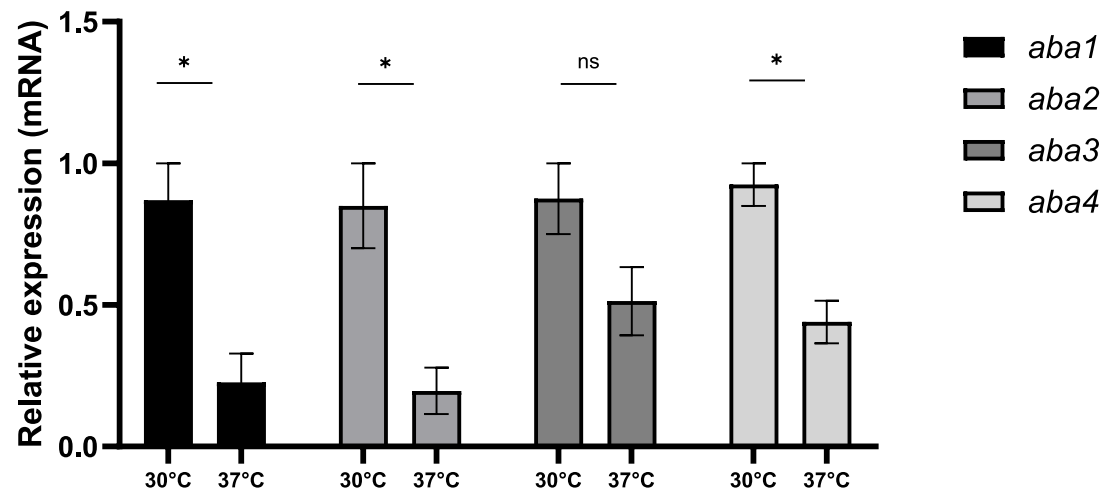

Supplement: Multimedia component 8 [file mmc8.pdf]

A

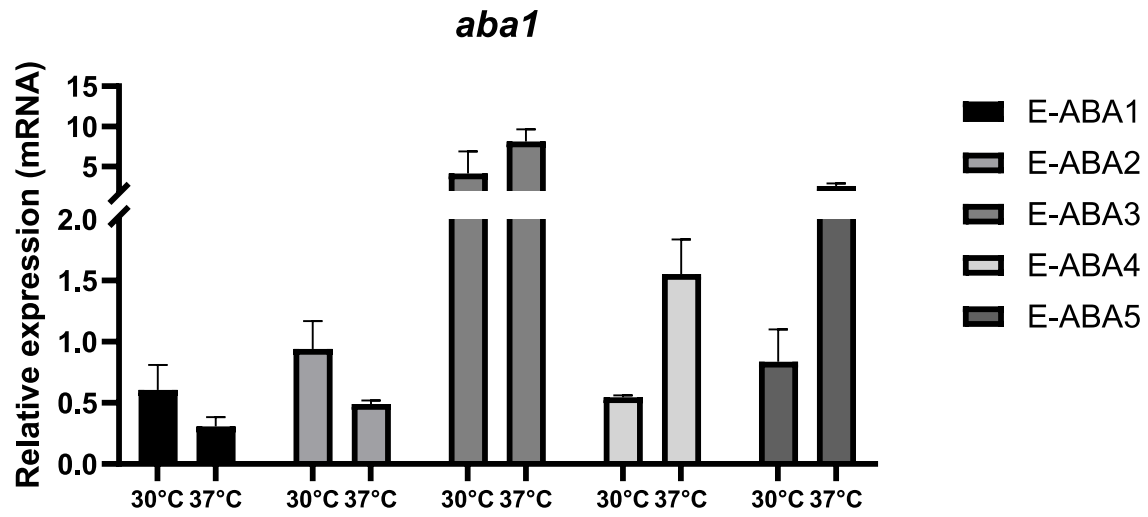

B

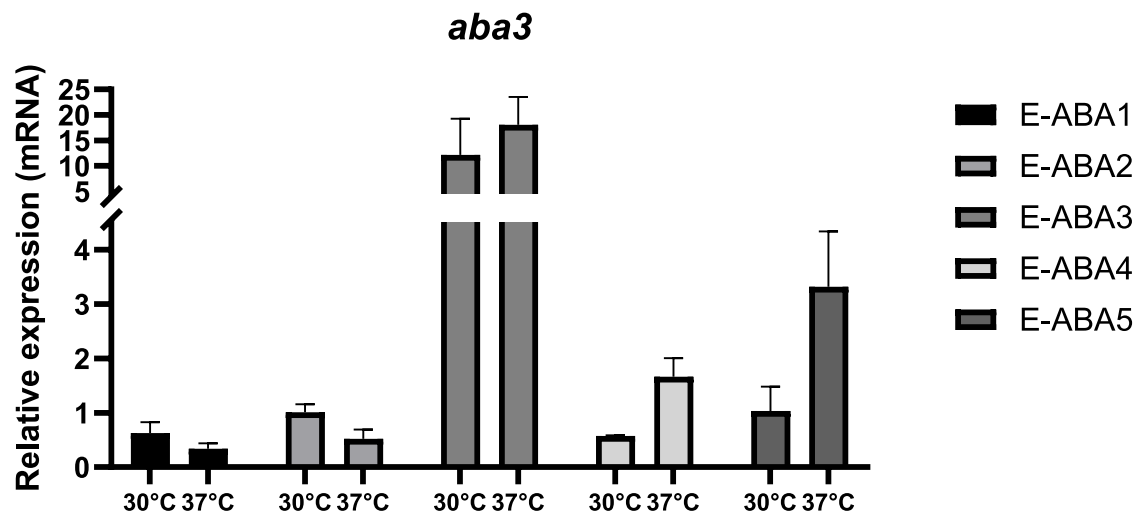

C

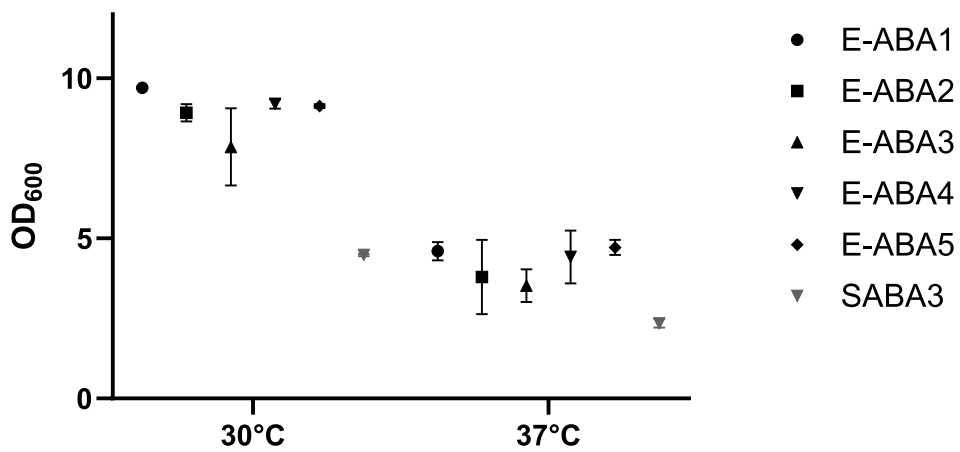

Supplement: Multimedia component 9 [file mmc9.pdf]
